# Pan-cancer analysis implicates novel insights of lactate metabolism into immunotherapy response prediction and survival prognostication

**DOI:** 10.1186/s13046-024-03042-7

**Published:** 2024-04-25

**Authors:** Dongjie Chen, Pengyi Liu, Xiongxiong Lu, Jingfeng Li, Debin Qi, Longjun Zang, Jiayu Lin, Yihao Liu, Shuyu Zhai, Da Fu, Yuanchi Weng, Hongzhe Li, Baiyong Shen

**Affiliations:** 1 Department of General Surgery, Pancreatic Disease Center, Ruijin Hospital, Shanghai Jiao Tong University School of Medicine, Shanghai, 200025 China; 2 Research Institute of Pancreatic Diseases, Shanghai Key Laboratory of Translational Research for Pancreatic Neoplasms, Shanghai Jiao Tong University School of Medicine, Shanghai, 200025 China; 3 State Key Laboratory of Oncogenes and Related Genes, Institute of Translational Medicine, Shanghai Jiao Tong University, Shanghai, 200025 China; 4 Department of General Surgery, Shanghai General Hospital, Shanghai Jiao Tong University School of Medicine, Shanghai, 200080 China; 5 Department of General Surgery, Taiyuan Central Hospital, Taiyuan, Shanxi 030009 China

**Keywords:** Lactate metabolism, Immunotherapy, Survival prognostication, Pan-cancer analysis, scRNA-seq

## Abstract

**Background:**

Immunotherapy has emerged as a potent clinical approach for cancer treatment, but only subsets of cancer patients can benefit from it. Targeting lactate metabolism (LM) in tumor cells as a method to potentiate anti-tumor immune responses represents a promising therapeutic strategy.

**Methods:**

Public single-cell RNA-Seq (scRNA-seq) cohorts collected from patients who received immunotherapy were systematically gathered and scrutinized to delineate the association between LM and the immunotherapy response. A novel LM-related signature (LM.SIG) was formulated through an extensive examination of 40 pan-cancer scRNA-seq cohorts. Then, multiple machine learning (ML) algorithms were employed to validate the capacity of LM.SIG for immunotherapy response prediction and survival prognostication based on 8 immunotherapy transcriptomic cohorts and 30 The Cancer Genome Atlas (TCGA) pan-cancer datasets. Moreover, potential targets for immunotherapy were identified based on 17 CRISPR datasets and validated via in vivo and in vitro experiments.

**Results:**

The assessment of LM was confirmed to possess a substantial relationship with immunotherapy resistance in 2 immunotherapy scRNA-seq cohorts. Based on large-scale pan-cancer data, there exists a notably adverse correlation between LM.SIG and anti-tumor immunity as well as imbalance infiltration of immune cells, whereas a positive association was observed between LM.SIG and pro-tumorigenic signaling. Utilizing this signature, the ML model predicted immunotherapy response and prognosis with an AUC of 0.73/0.80 in validation sets and 0.70/0.87 in testing sets respectively. Notably, LM.SIG exhibited superior predictive performance across various cancers compared to published signatures. Subsequently, CRISPR screening identified LDHA as a pan-cancer biomarker for estimating immunotherapy response and survival probability which was further validated using immunohistochemistry (IHC) and spatial transcriptomics (ST) datasets. Furthermore, experiments demonstrated that LDHA deficiency in pancreatic cancer elevated the CD8^+^ T cell antitumor immunity and improved macrophage antitumoral polarization, which in turn enhanced the efficacy of immunotherapy.

**Conclusions:**

We unveiled the tight correlation between LM and resistance to immunotherapy and further established the pan-cancer LM.SIG, holds the potential to emerge as a competitive instrument for the selection of patients suitable for immunotherapy.

**Supplementary Information:**

The online version contains supplementary material available at 10.1186/s13046-024-03042-7.

## Introduction

The advent of immunotherapy marks a transformative epoch in cancer treatment, yielding unprecedented clinical advantages for patients [1]. Nevertheless, limited response rates and the inability to predict clinical efficacy obstacle their further application, which underscores the imperative need for biomarker detection to facilitate precise medicine and formulate effective combination strategies against immune resistance [2]. Conventional biomarker screening is predominantly centered on the exploration of omics data derived from patients [3–6]. However, this approach solely captures the mean genetic expression within a heterogeneous cell population, leading to limited predictive values of pre-existing immunotherapy biomarkers from these studies. The advantage of single-cell RNA sequencing (scRNA-Seq) and spatial transcriptomics (ST) enabled us to detect expression profiles of the transcriptome at a single-cell and spatial resolution which in turn unveils novel targets with superior performance [7, 8].

Lactate, an intermediate metabolite of the Warburg effect which induces an acidic tumor microenvironment (TME) through the utilization of glucose for glycolysis initially proposed by Otto Warburg in the 1920s [9]. This phenomenon, commonly referred to as aerobic glycolysis, was initially attributed solely to the elevated glucose consumption of cancer cells. However, mounting evidence indicates that immune cells also exhibit this metabolic behavior [10, 11]. Notably, these cells enhance proliferative activity and establish an immunosuppressive phenomenon due to lactate, conferring upon them unlimited potential for immune escape [12]. Lactic acid has been demonstrated to impede the production of IFN-γ, granzyme B, and perforin in T cells and NK cells, along with inhibiting proliferation, thereby compromising cytotoxic responses [13–15]. Although the accumulation of intratumoral lactic acid proves detrimental to anti-tumor immunity, it is imperative to recognize that evolutionary pressures have influenced the adaptive mechanisms of immune cells to these elements. Analogous to checkpoint molecules like PD-1 and CTLA-4, which ensure self-tolerance and mitigate excessive tissue damage resulting from hyperactive immune responses, suppression induced by an acidic, lactate-rich environment can be construed as a physiologically relevant metabolic checkpoint. Within the context of tumors, targeting these maladapted programs holds promise as a therapeutic approach [16]. Hence, lactate could serve as a mediator connecting metabolic reprogramming to immunosuppression [17]. Previous studies have developed biomarkers based on lactate metabolism (LM) in predicting immunotherapy response in breast cancer [18], lung cancer [19] and kidney renal clear cell carcinoma [20], but direct research demonstrating the negative relationship between LM and immunotherapy response at both pan-cancer levels is lacking. With the assistance of the scRNA-seq technique and multiple machine learning (ML) algorithms, we can precisely characterize LM and determine the LM-related signature at the pan-cancer level to unveil the influence of LM in immunotherapy response.

**Fig. 1:**
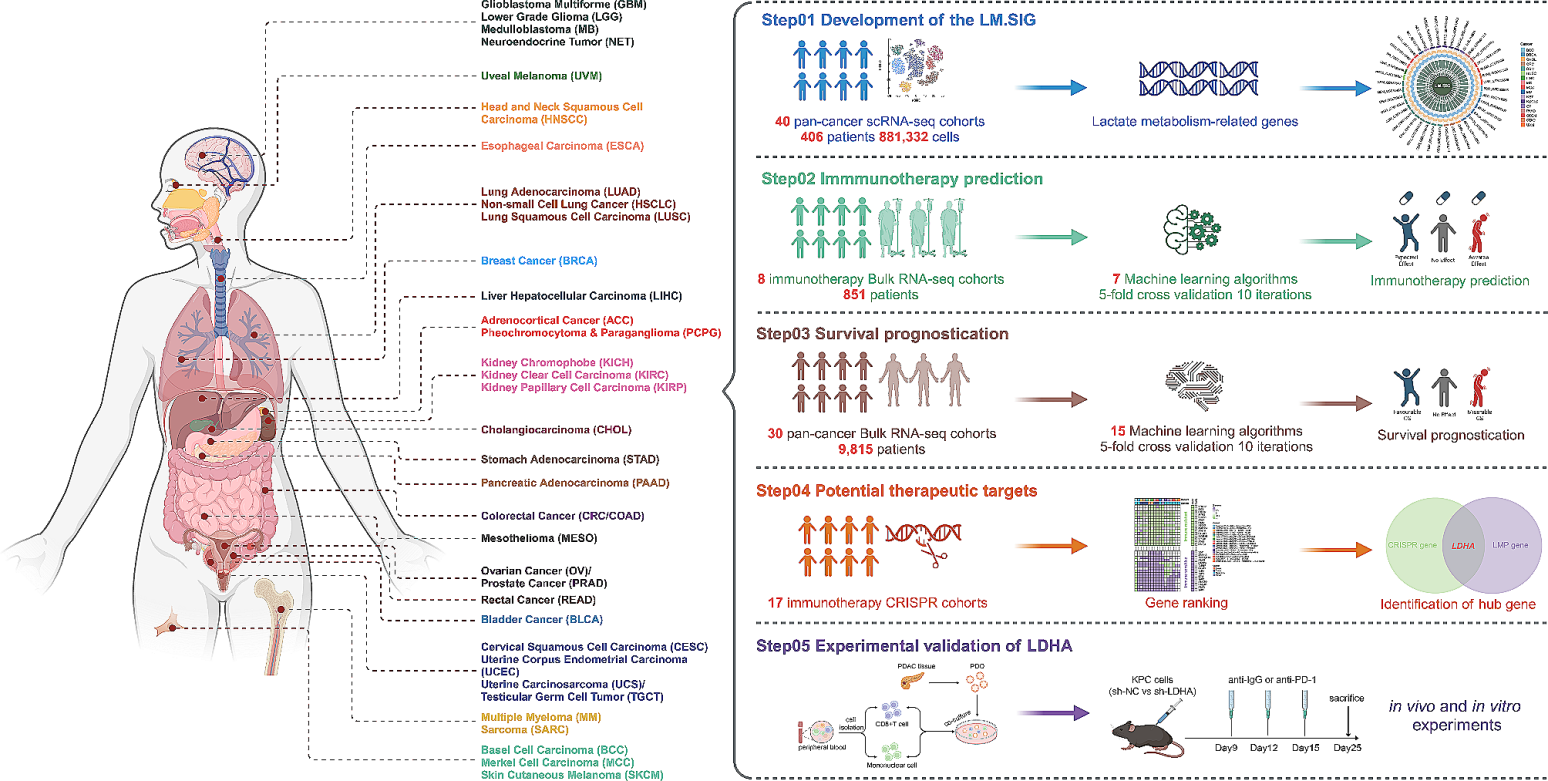
The flowchart of this study

Herein, we first illustrated and validated the inverse relationship between LM and immunotherapy response in two immunotherapy scRNA-seq datasets. Subsequently, LM-related signature (LM.SIG) was established based on 40 pan-cancer scRNA-seq cohorts containing 406 patients and 881,332 cells across 17 types of cancer. The predictive performance of LM.SIG in immunotherapy response was further investigated and verified via 7 ML algorithms based on 8 pan-cancer immunotherapy bulk RNA-seq cohorts (including 851 patients). Besides, 15 survival-specific ML algorithms in the SurvBenchmark design [21] were applied to characterize the survival prognostication of LM.SIG through an integrated analysis of pan-cancer The Cancer Genome Atlas (TCGA) RNA-seq cohorts (including 30 types of cancer). Finally, we identified the LDHA as the most potential target from LM.SIG was based on 17 CRISPR datasets, and validated its capacity in pancreatic cancer (PC) via experiments. The flowchart of this study is shown in Fig. 1.

## Materials and methods

### Cell culture and transfection

KPC1199 cell line was cultured in DMEM supplemented with 10% FBS (Gbico) and 1% P/S (NCM). Cells were cultured at 37 °C in an atmosphere containing 5% CO2. Lentiviruses were sourced from Bioegene (Shanghai, China). For stable transfections, the appropriate lentivirus was added to the supernatant, and the medium was changed after 6 to 8 h. Following expression verification, cells were treated with 2 µg/mL puromycin to identify cells expressing the resistance gene, which represented stably transfected cell lines.

### Patient-derived organoids (PDOs) construction and measurement

The samples were obtained from Ruijin Hospital, Shanghai Jiao Tong University School of Medicine. The study protocol was approved by the Research Ethics Committee of Ruijin Hospital, School of Medicine, Shanghai Jiao Tong University. All enrolled participants consented to attend this cohort study and signed written informed consent. PC tumor tissues from patients were promptly dissected into small pieces in precooled RPMI-1640 and digested using the human Tumor Dissociation Kit (Miltenyi) at 37 °C for 20 min. Afterward, filtration through the Falcon® 40 μm Cell Screen (Corning) was completed, and single cells were seeded into Matrigel (Corning), wherein they were subsequently cultured in a complete organoid culture medium, OmaStem® Pan-cancer Advanced (OmaStem). The relative activity of the organoids was measured using the CellTiter-Glo® 3D Cell Viability Assay (Promega) according to the manufacturer’s instructions. Lactate acid in supernatant was measured through L-lactic acid (L-LA) content test kit (Solarbio) following the instructions. A characteristic absorption peak at 570 nm was detected to calculate L-LA content, depending on the standard curve.

### scRNA-seq and ST Immunotherapy datasets

2 immunotherapy scRNA-seq cohorts, GSE115978 (a melanoma scRNA-seq cohort, https://www.ncbi.nlm.nih.gov/geo/query/acc.cgi? acc=GSE115978) and GSE123813 (a basal cell carcinoma scRNA-seq cohort, https://www.ncbi.nlm.nih.gov/geo/query/acc.cgi? acc=GSE123813) were screened out to explore the correlation between lactate metabolism and immunotherapy response. Additionally, preprocessed ST data of tumor sections from 2 patients with hepatocellular carcinoma (HCC) receiving anti-PD-1 treatment (non-responders, *n* = 1; responders, *n* = 1) was extracted from Mendeley Data (skrx2fz79n) [22] (Table S1).

### Pan-cancer scRNA-seq datasets and processing

To develop a lactate metabolism-specific signature, 40 pan-cancer scRNA-seq data containing 406 patients and 881,332 cells were collected (Table S2). These large-scale scRNA-seq data included 17 types of cancer, namely Basel Cell Carcinoma (BCC), Breast Cancer (BRCA), Cholangiocarcinoma (CHOL), Colorectal Cancer (CRC), Glioblastoma Multiforme (GBM), Head and Neck Squamous Cell Carcinoma (HNSCC), Liver Hepatocellular Carcinoma (LIHC), Medulloblastoma (MB), Merkel Cell Carcinoma (MCC), Multiple Myeloma (MM), Neuroendocrine Tumor (NET), Non-small Cell Lung Cancer (NSCLC), Ovarian Cancer (OV), Pancreatic Adenocarcinoma (PAAD), Skin Cutaneous Melanoma (SKCM), Stomach Adenocarcinoma (STAD) and Uveal melanoma (UVM). Raw data of these datasets were extracted from Gene Expression Omnibus (GEO, https://www.ncbi.nlm.nih.gov/geo), The European Genome-phenome Archive (EGA, https://ega-archive.org), and Array Express (https://www.ebi.ac.uk/arrayexpress). Standard workflow for scRNA-seq was implemented via the R package “Seurat”. Low quality cells (< 500 genes/cell, > 5% mitochondrial genes or a log10(UMI per gene) < 3) were excluded. The “Seurat” package was applied for normalization and scaling of the expression matrix, using default settings. The expression matrix dimensionality was reduced through principal component analysis (PCA), focusing on 2,000 highly variable genes. Unsupervised cluster analysis was implemented and visualized via t-distributed Stochastic Neighbor Embedding (t-SNE) reduction. Cluster annotations were assigned based on canonical marker expression and references from the original literature (Table S2). Furthermore, differential genes within each cluster were pinpointed using the FindAllMarkers function, applying the criteria of logFC > 0.25, min.pct > 0.1, and adjusted *p*-value < 0.05.

### Pan-cancer transcriptomic datasets and processing

The pan-cancer cohort of batch effects normalized transcriptomic data of 9,815 patients with complete survival information in 30 TCGA cohorts was extracted from the UCSC database (https://xenabrowser.net). Three types of cancer, diffuse large B cell lymphoma (DLBC), acute myeloid leukemia (LAML), and thymoma (THYM) were excluded on account of abundant immune cells [23]. The total tumor mutation burden (TMB) data for pan-cancer analysis from TCGA cohorts was acquired through the cBioPortal (https://www.cbioportal.org). Additionally, transcriptomic and clinical data of 4,434 patients in 10 cohorts was collected as testing sets to evaluate the prognostic efficacy of the prognostic model associated with LM, including E-MTAB-6134 (pancreatic cancer, *n* = 288), CGGA-693 (glioma, *n* = 657), CGGA-301 (glioma, *n* = 285), METABRIC (breast cancer, *n* = 1935), prad-su2c-2019 (prostate cancer, *n* = 71), GSE13507 (bladder cancer, *n* = 165), GSE17538 (colorectal cancer, *n* = 232), GSE30219 (lung cancer, *n* = 274), GSE72094 (lung cancer, *n* = 398) and GSE138866 (ovarian cancer, *n* = 129). All raw data of the above datasets were downloaded from GEO and cBioPortal. The expression matrix underwent z-score normalization across all transcriptomic datasets to ensure standardization. The details of these cohorts are summarized in Table S3.

### Bulk RNA-seq immunotherapy datasets

Transcriptomic profile and clinical information of immunotherapy-treated samples from 10 immunotherapy bulk RNA-Seq datasets were collected to assess the predictive performance of LM.SIG, including 5 SKCM cohorts (Liu SKCM [24], Gide SKCM [25], Riaz SKCM [26], Hugo SKCM [27] and Van SKCM [28]), 1 Renal Cell Carcinoma (RCC) cohort (Bruan RCC [29]), 1 Urothelial Carcinoma (UC) cohort (Mariathasan UC [30]), 1 GBM cohort (Zhao GBM [31]), 1 Gastric Cancer (GC) cohort (Kim GC [32]) and 1 NSCLC cohort (Jung NSCLC [33]). The relevant processed data of these datasets were sourced from the related published articles (Table S4).

### Published signatures for comparison

To evaluate the performance of LM.SIG in predicting immunotherapy response, the following 13 published immunotherapy response signatures were collected for comparison: 6 pan-cancer signatures (Ayers.INFG.SIG [34], Ayers.T.cell.SIG [34], Topalian.PDL1.SIG [35], Dominguez.CAF.SIG [36], Ju.NLRP3.SIG [37] and Rooney.Cytotoxic.SIG [38]) and 7 melanoma-specific signatures (Shukla.CRMA.SIG [39], Auslander.IMPRES.SIG [6], Hugo.IPRES.SIG [27], Jerby-Arnon.TcellExc.SIG [40], Xiong.ImmmunCells.SIG [41], Cui.IMS.SIG [42], and Yan.TRS.SIG [43]). Similar algorithms of these signatures were applied according to original articles, and the area under curve (AUC) value was further conducted to assess their fitting capability.

### CRISPR datasets processing

Our study incorporated 17 reorganized datasets derived from 7 well-established CRISPR/Cas9 libraries, each pertaining to the individual impact of gene knockout on tumor immunity. These studies, conducted by Freeman [44], Kearney [45], Manguso [46], Pan [47], Patel [48], Vredevoogd [49] and Lawson [50], encompass diverse cancer types, namely SKCM, BRCA, CRC, and RCC cell lines. We collected the first 6 studies from Fu et al. [51], and augmented our dataset with an additional CRISPR cohort from Lawson et al. [50]. Based on distinct cell lines and treatment conditions, we reorganized this study into 17 datasets (Table S5). Our comprehensive CRISPR analysis aimed to identify genes with a heightened likelihood of modulating anti-tumor cytotoxicity and affecting immunotherapy response among diverse cohorts. Log-fold changes (logFCs) in small guide RNA (sgRNA) reads were computed between with CTLs vs. without CTLs or immune-competent vs. immune-deficient subgroups and served as a measuring tool to assess the cancer fitness after gene knockout in the presence of anti-tumor immunity. logFCs of all CRISPR datasets were normalized as z-scores to remove the batch effects and facilitate gene comparisons. A more robust immune response is observed with lower z-scores following gene knockout. Specifically, genes ranked at the top based on their z-scores are identified as exhibiting resistance to immune-related processes.

### Pathway analysis and anti‑tumor immunity evaluation

Gene set variation analysis (GSVA) was performed to quantify the pathway-specific scores across the pan-cancer scRNA-seq and bulk RNA-seq datasets via the “GSVA” R package. The background gene sets (LM-related gene set and HALLMARK gene sets) were downloaded from the Molecular Signatures Database (MSigDB) database (https://www.gsea-msigdb.org/gsea/msigdb) (Table S6). Pathway enrichment analysis was conducted via the R package “clusterProfiler” based on the Reactome Knowledgebase (https://reactome.org), Gene Ontology (GO) terms and Kyoto Encyclopedia of Genes and Genomes (KEGG) database. In pan-cancer TCGA cohorts, we conducted a comprehensive assessment of the relationship between LM.SIG and tumor-infiltrating leukocytes (TILs) as well as immune-related genes which were sourced from Thorsson et al [52]. For the quantification of immune cell infiltration, the R package “MCPcounter” was applied.

### Generation of a predictive model for immunotherapy response

In order to appraise the capacity of LM.SIG in predicting the immunotherapy response, 8 immunotherapy cohorts mentioned above were obtained. 5 cohorts (Bruan RCC, Mariathasan UC, Liu SKCM, Gide SKCM and Riaz SKCM) with the most number of patients were merged as a meta-cohort (*n* = 772). The “ComBat” method was implemented to remove batch effects based on the R package “sva”. Then, we categorized the patients receiving immunotherapy into training set (*n* = 618) and validation set (*n* = 154) with a ratio of 8:2, the other 3 cohorts were listed as testing sets (*n* = 79). 7 ML algorithms, including support vector machine (SVM), Naïve Bayes (NB), random forest (RF), k-nearest neighbors (KNN), AdaBoost Classification Trees (AdaBoost), boosted logistic regressions (LogiBoost), and cancerclass were applied to train immunotherapy response classification model based on LM.SIG. 5-fold cross-validation was adopted for tuning. For each single resampling, 10 iterations were employed for robustness. Except for the cancerclass using the R package “cancerclass”, other ML algorithms were trained and predicted via R package “caret”. The LM.SIG model, exhibiting optimal performance in the validation set, was selected as the ultimate model for further analysis. The 3 independent testing sets and 6 published signatures mentioned above were collected to examine the predictive ability of the final model.

### Derivation of LM‑related prognostic model

SurvBenchmark, a benchmarking design for survival models, was applied to investigate the prognostic value of LM.SIG [21]. This design not only concentrates on classical approaches but also evaluates state-of-the-art ML survival models. A total of 9,815 pan-cancer TCGA patients were randomly divided into training set (80%, *n* = 7865) and validation set (20%, *n* = 1950). Besides, 4,434 patients of 10 pan-cancer datasets were obtained as a testing set. The following 15 algorithms in the SurvBenchmark study were implemented: Lasso_Cox, Ridge_Cox, Elastic net cox (EN_Cox), Random survival forest (RSF), Multi-task logistic regression (MTLR), Deep learning survival model (DNNSurv), CoxBoost, Cox model with genetic algorithm as feature selection method (Cox_GA), Multi-task logistic regression model with genetic algorithm as feature selection method (MTLR_GA), Boosting cox model with genetic algorithm as feature selection method (CoxBoost_GA), Multi-task logistic regression model with ranking based method as feature selection method (MTLR_DE), Boosting cox model with ranking based method as feature selection method (CoxBoost_DE), Survival support vector machine (SurvivalSVM), DeepSurv, DeepHit. We trained the model on the training set and calculated the evaluation metrics using the validation and testing set with 10 iterations repeated 5-fold cross-validation. The final model was identified with the best AUC/time-dependent AUC, and concordance index (C-index), including Harrell’s C-index [53], Begg’s C-index [54], Uno’s C-index [55], and GH C-index [56]. More detailed information is listed in Table S7.

### Primary immune cells extraction and co-culture

Peripheral blood of patients was stored in anticoagulated tubes, diluted with PBS, and lightly spread over Lymphocyte Separation Medium (YEASEN). Peripheral Blood Mononuclear Cells (PBMCs) were obtained by centrifugation using density gradient centrifugation for 25 min. Anti-human CD8 Microbeads (Miltenyi) and anti-human CD14 Microbeads (Miltenyi) and MACS® MultiStand (Miltenyi) were employed for T cells and Mononuclear cells sorting, respectively. T cells were activated by CD3/CD28, and Mononuclear cells were active by M-CSF. Activated T cells and macrophages were co-cultured with sh-NC or sh-LDHA PC PDOs.

### RNA extraction and real-time quantitative PCR (RT-qPCR)

RNA was extracted from cells using the SteadyPure Universal RNA Extraction Kit (Accurate Biology) and then reverse transcribed to cDNA using the Evo M-MLV reverse transcription kit (Accurate Biology). Post-transcription, the concentration and purity of the RNA were determined. Relative RNA expression levels were detected using the Evo M-MLV One-Step RT-qPCR Kit (SYBR) on qTOWER384G (Analytik Jena). The forward and reverse primers are listed in Table S8.

### Western blotting

Proteins were transferred to polyvinylidene difluoride (PVDF) membranes (Merck Millipore, USA) after separating cell lysates using 10% sodium dodecyl sulfate-polyacrylamide gel electrophoresis (SDS-PAGE). Primary antibodies were applied to the membranes overnight at 4 °C, followed by incubation with secondary antibodies. Target proteins were detected using the Imaging System (Tanon, China). The antibodies employed were as follows:

β-Actin Rabbit mAb (1:5000, Abclonal, #AC048).

LDHA Rabbit mAb (1:1000, Cell Signaling Technology, #3558S).

### Flow cytometry and intracellular staining

Cells were harvested, centrifuged, washed, and suspended in 100 µL pre-cooled 1% BSA solution (dissolved in PBS). They were then stained with flow antibodies conjugated with the indicated fluorescence for half an hour by following the recommended concentration in the dark. The cytokines and intracellular proteins were detected according to the manufacturer’s instructions of Cytofix/Cytoperm Fixation and Permeabilization Solution (BD Bioscience). CytoFLEX S (Beckman) recorded corresponding fluorescence signals after unbound antibodies were discarded. Various groups, including a blank group, a single antibody-stained group, and a sample group, were used for voltage adjustment and compensation.

### Animal studies

6-week-old male C57BL/6 were selected for in vivo study. Approximately 2*10^6^ KPC cells (sh-NC and sh-LDHA) resuspended in 150ul PBS were injected into the lateral abdomen of mice. Three intraperitoneal injections of anti-PD1 (10 mg/kg) or anti-IgG (10 mg/kg) were given on days 9, 12 and 15, respectively. Tumor volume was measured every 4 days starting from day 7. 25 days later, the mice were euthanized, and the subcutaneous tumors were collected, photographed, weighed, and stained with hematoxylin and eosin (H&E) for IHC analysis.

### H&E and IHC

Tissues were formalin-fixed, paraffin-embedded, and sectioned onto slides. IHC staining was thereupon performed using the standard streptavidin-biotin-peroxidase complex method. Following deparaffinization and rehydration, the slides were successively subjected to antigen retrieval, inactivation, incubation with primary and secondary antibodies, DAB staining, and sealing. Finally, representative pictures were captured under a microscope.

### Statistical analysis

R v4.2.2 was applied to conduct all statistical analyses in this study. The Wilcox test was implemented to compare the GSVA scores of LM between two different subgroups. Spearman correlation analysis was used to investigate the relationship between LM.SIG and hallmark pathways or immune characteristics. The false discovery rate (FDR) was calculated by the Benjamini-Hochberg procedure (B-H). The log-rank test was utilized to assess the significance of observed differences in overall survival (OS). Statistical significance was determined by a two-tailed *p*-value less than 0.05, unless explicitly specified otherwise.

## Results

### Upregulated LM is associated with resistance to immunotherapy

**Fig. 2:**
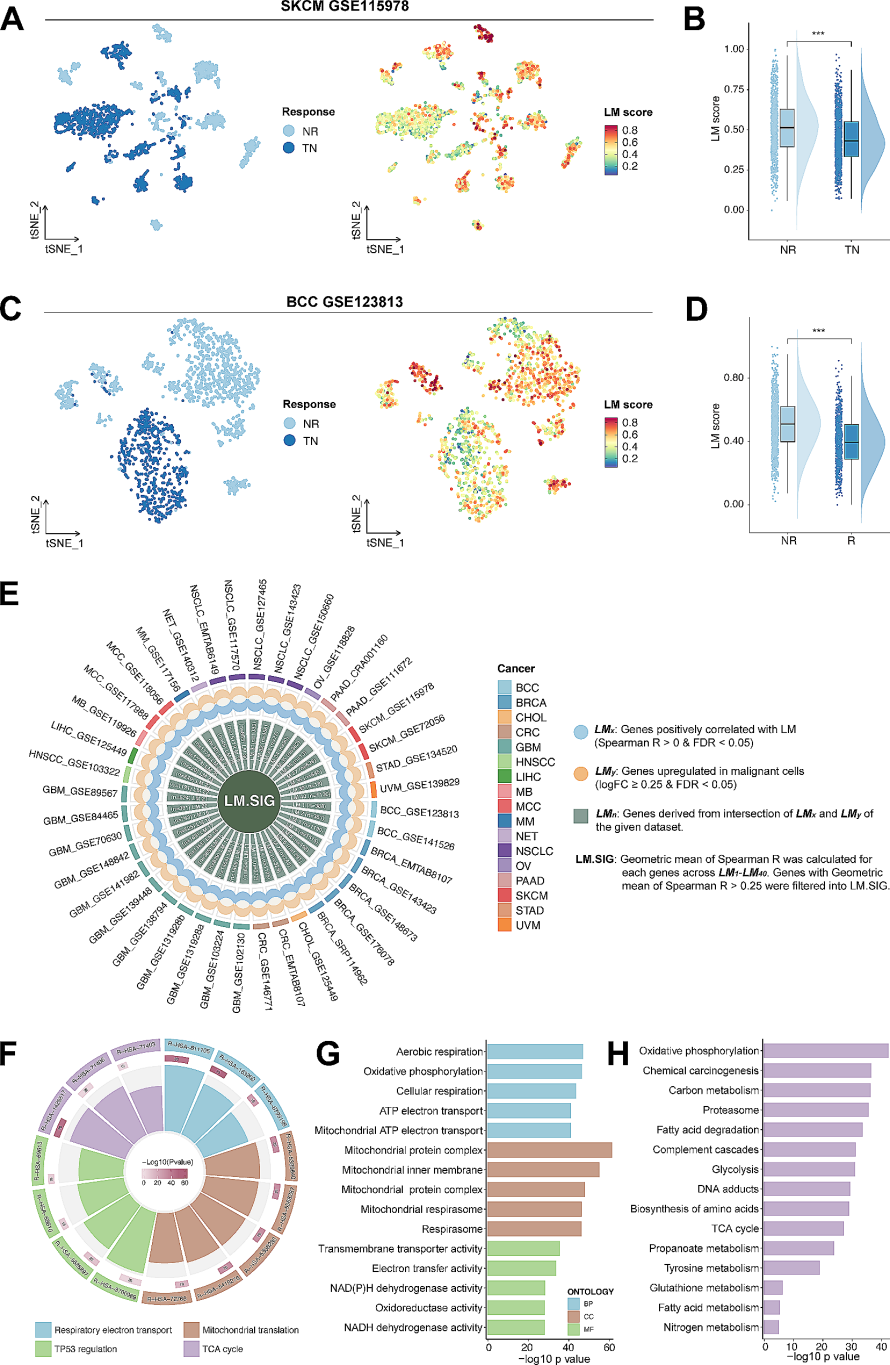
Development of the LM.SIG. tSNE plot of malignant cells from SKCM (**A**) and BCC (**C**). tSNE plots in the right panel revealed the distribution of the GSVA score of LM-related gene sets (LM score). Boxplot depicting the distribution of the LM score in NR and R/TN patients from SKCM (**B**) and BCC (**D**). (**E**) Circos plot depicting the development of LM.SIG. The outer circle represents the enrolled 40 pan-cancer scRNA-seq datasets. The middle circle represents the veen plot depicting the intersection of *LM*_*x*_ (blue) and *LM*_*y*_ (brown). *LM*_*x*_ represents genes positively correlated with LM and *LM*_*y*_ represents genes upregulated in malignant cells. The inner circle represents *LM*_*n*_. The final LM.SIG was established based on the geometric mean of Spearman’s correlation coefficient (R) computed for individual genes spanning *LM*_*1*_–*LM*_*40*_. Pathway enrichment analysis of LM.SIG based on Rectome (**F**), GO (**G**) and KEGG (**H**) databases. (ns, not significant; *p < 0.05, **p < 0.01, ***p < 0.001, ****p < 0.0001)

To explore the relationship between LM and immunotherapy response, 2 immunotherapy scRNA-seq cohorts (GSE115978, GSE123813) were collected. We computed the LM scores of cells in these cohorts based on the LM-related genes (Table S6) as previously mentioned via GSVA analysis. After excluding patients without malignant cells, 11 non-responders (NR) and 13 treatment-naïve (TN) SKCM patients in GSE115978 were enrolled. Due to the lack of responders (R) in this dataset, TN patients (probably including both potential R and NR) were acquired for comparison. As shown in Fig. 2A, malignant cells with elevated LM levels demonstrated a propensity for enrichment within the NR subgroup. Subsequent analysis revealed the elevated LM in NR patients (Fig. 2B, *p* < 0.001). Another BCC immunotherapy scRNA-seq cohort (including 4 NR and 6 R) further validated this result (Fig. 2C-D, *p* < 0.001). Together, these results indicate that LM is associated with resistance to immunotherapy.

### Establishment of LM.SIG using pan‑cancer scRNA-seq cohort

As LM is considerably correlated with the resistance to anti-tumor immunity, we suggested that an LM.SIG representing the LM infiltration of the cancer might facilitate the effectiveness of immunotherapy prediction. Hence, 40 pan-cancer scRNA-seq datasets were screened out to establish the LM.SIG (Fig. 2E, Table S2). First, Spearman correlation analysis was conducted between GSVA scores (based on the LM-related genes) and gene expression for malignant cells in pan-cancer scRNA-seq cohorts. We have designated the term *LM*_*x*_ to denote genes exhibiting a positive correlation with GSVA scores (*R* > 0, FDR < 0.05). Besides, genes significantly upregulated in malignant cells were deemed as *LM*_*y*_ (logFC ≥ 0.25, FDR < 0.05). Then, *LM*_*x*_ (Table S9) and *LM*_*y*_ (Table S10) were intersected into *LM*_*n*_ (*n* = 1–40) (Table S11) for each scRNA-seq cohort to obtain up-regulated tumor-specific genes that were positively associated with LM. The geometric mean of Spearman’s correlation coefficient (R) was computed for individual genes spanning *LM*_*1*_–*LM*_*40*_, and genes exhibiting a geometric mean of Spearman’s R exceeding 0.25 were filtered into the LM.SIG, which contained 84 genes (Table S12). The underlying functional exploration was implemented based on Reactome, GO and KEGG analysis. Results illustrated that LM.SIG is mainly enriched in lactate metabolism-related and tumorigenic pathways, such as Nicotinamide adenine dinucleotide(NADH)-related pathways, tricarboxylic acid (TCA) cycle-related pathways (Fig. 2F-H) and TP53 regulation (Fig. 2F). Among those 84 genes, several genes have been reported to be related to immune activity, such as C1QBP [57], TUFM [58], LDHA [59], LDHB [60] and ACAT1 [61].

### Immune atlas of LM.SIG based on pan‑cancer TCGA cohort

**Fig. 3:**
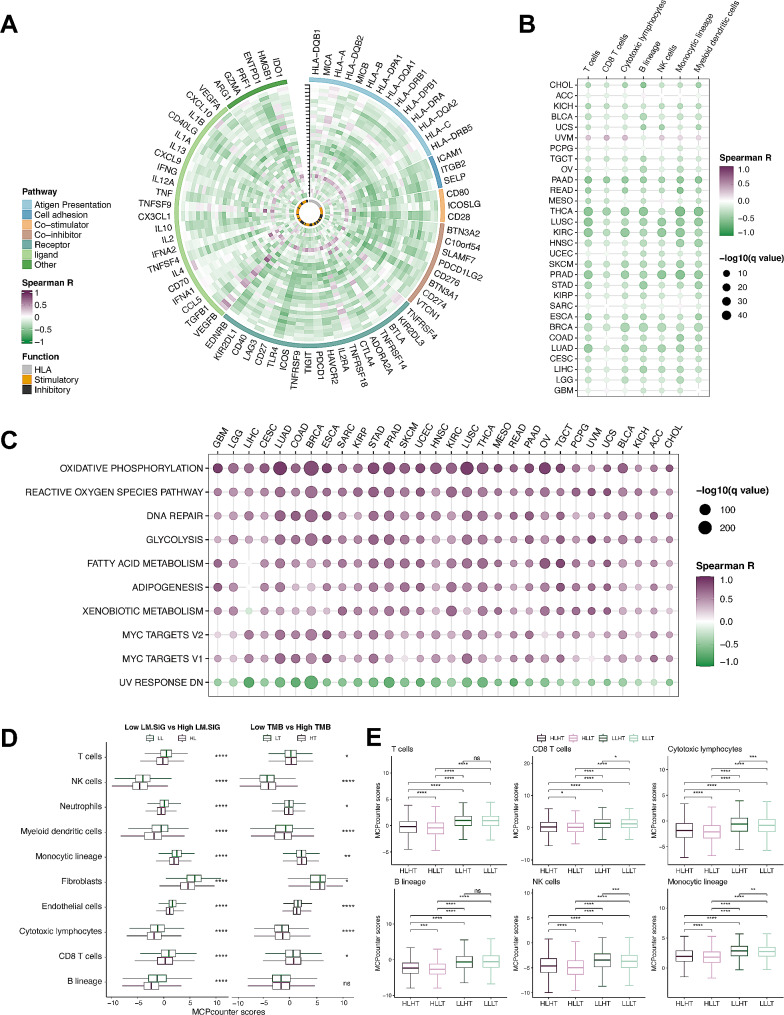
Immune atlas of LM.SIG. (**A**) Circos plot illustrating the correlation between LM.SIG and the expression levels of immune-related genes across diverse cancer types. (**B**) Heatmap illustrating the correlation between LM.SIG and the infiltration of immune cells across diverse cancer types. (**C**) Heatmap illustrating the correlation between LM.SIG and the top 10 hallmark pathways across diverse cancer types. (**D, E**) Boxplots depicting the correlation of immune cell infiltration with LM.SIG and TMB. (ns, not significant; *p < 0.05, **p < 0.01, ***p < 0.001, ****p < 0.0001)

In TCGA pan-cancer datasets, GSVA analysis was applied to calculate the LM.SIG score based on 84 LM.SIG genes. To further investigate the potential correlation between LM.SIG score and immune suppression, we carried out a comprehensive analysis based on 75 immune-related genes [52]. As shown in Fig. 3A, a significantly negative correlation was detected between LM.SIG score and expression of these genes across pan-cancer datasets. Meanwhile, the abundance of immune cells was appraised via the R package “MCPcounter” [62] and tumors with high LM.SIG score earned decreased infiltration of anti-tumor immune cells, including NK cells and cytotoxic lymphocytes (Fig. 3B). Then, we calculated the correlation analysis between GSVA scores of the hallmark gene set and LM.SIG to dig into whether immunosuppressive pathways were enriched in high LM.SIG score tumors. Top-ranked pathways, such as oxidative phosphorylation, reactive oxygen species pathway, DNA repair and MYC target pathway, were confirmed to be upregulated in patients with high LM.SIG score (Fig. 3C).

Moreover, we performed a series of analyses between LM.SIG and TMB. Based on the median LM.SIG score and TMB across TCGA pan-cancer cohorts, patients were divided into 8 subgroups for comparison: High LM.SIG (HL), Low LM.SIG (LL), High TMB (HT), Low TMB (LT), High LM.SIG / High TMB (HLHT), High LM.SIG / Low TMB (HLLT), Low LM.SIG / High TMB (LLHT), Low LM.SIG / Low TMB (LLLT). High LM.SIG represents a miserable immune response while high TMB adopts the converse stance. As anticipated, reduced infiltration of cytotoxic lymphocytes was found in the HL (*p* < 0.001) and LT (*p* < 0.001) subgroups (Fig. 3D). Integrated analysis also demonstrated that LLHT was enriched in the highest abundance of cytotoxic lymphocytes whereas HLLT obtained the lowest (Fig. 3E). Hence, the interaction of High LM.SIG and Low TMB (HLLT) may lead to a cytotoxic lymphocyte-deficient tumor immune microenvironment (TIME). Conversely, abundant cytotoxic lymphocytes were found to be enriched in the LLHT subgroup. However, the immunological characteristics of HLHT and LLLT appeared to be more controversial than those of HLLT and LLHT. This tendency arose from the presence of both immune-deficient (HL or LT) and immune-competent (LL or HT) factors in HLHT and LLLT. In summary, the capacity of anti-tumor immunity, ranked from top to bottom is as follows: LLHT > LLLT > HLHT > HLLT (Fig. 3E). Consequently, patients obtained lower LM.SIG tended to acquire better anti-tumor immunity compared to higher LM.SIG patients.

### Immunotherapy response prediction by LM.SIG

**Fig. 4:**
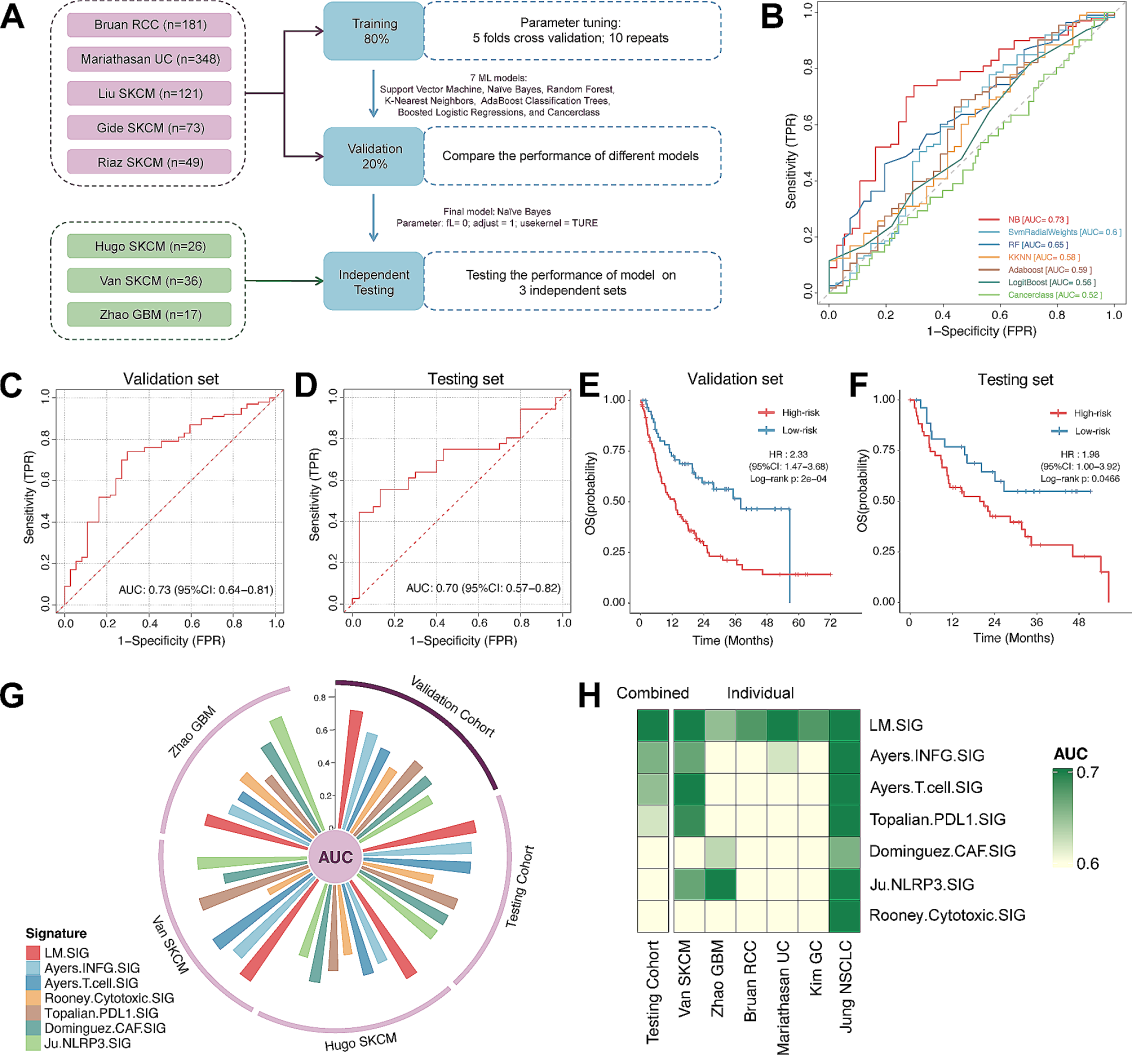
Immunotherapy response prediction of LM.SIG. (**A**) Flow chart of training, validating, and testing the LM.SIG model constructed via ML algorithms. (**B**) Comparison of multiple ROC plots depicting the performance of different ML algorithms in the validation set. ROC plot depicting the performance of the LM.SIG model in validation (**C**) and testing (**D**) cohort. Kaplan‑Meier curves comparing OS between High‑risk and Low‑risk patients in validation (**E**) and testing set (**F**). NR and R predicted by the LM.SIG model was defined as High‑risk and Low‑risk patients respectively. (**G**) Circos plot depicting the performance of other pan‑cancer signatures in the testing set. (**H**) Heatmap comparing the predictive value of LM.SIG and other pan‑cancer signatures

Given the tight relationship between LM.SIG and anti-tumor immunity, we hypothesized that whether LM.SIG could predict the response to immunotherapy. Based on the established LM.SIG, 8 immunotherapy cohorts with complete clinical information were compiled and categorized into training set (*n* = 618), validation set (*n* = 154) and testing set (*n* = 79) (Fig. 4A). We first trained the model with 7 ML methods and iterated 10 iterations repeated 5-fold cross-validation. The AUC values of the validation and testing sets were calculated. As shown in Fig. 4B-C, Naïve Bayes reached the highest AUC value of 0.73 in the validation set and was identified as optimal LM.SIG model. To further examine the robustness of the LM.SIG, the same algorithm was performed in the testing set and the AUC value was 0.70 (Fig. 4D). Next, we classified patients into High-risk (predicted NR) and Low-risk (predicted R) subgroups for survival analysis. The Kaplan-Meier plot showed that the Low-risk subgroup obtained favorable OS in the validation and testing sets (Fig. 4E-F). The following subgroup analysis was performed in the 3 testing sets and AUC ranged from 0.65 to 0.75 across these datasets (Figure S1).

Published articles have revealed numerous predictive signatures related to immunotherapy prediction. We compared the predictive value of LM.SIG with these signatures (including Ayers.INFG.SIG [34], Ayers.T.cell.SIG [34], Topalian.PDL1.SIG [35], Dominguez.CAF.SIG [36], Ju.NLRP3.SIG [37], and Rooney.Cytotoxic.SIG [38]). As shown in Fig. 4G, LM.SIG achieved the highest AUC of 0.73 in the validation set while the Rooney.Cytotoxic.SIG had an AUC of 0.47. Notably, most published signatures achieved higher stability merely in one or two datasets but performed far from satisfactory in other external cohorts, which may be due to the poor generalisability. For instance, the AUC of Ju.NLRP3.SIG achieved 0.74 in Zhao GBM and 0.67 in Van SKCM, but it ranged from 0.46 to 0.60 in other cohorts (Table S13). Conversely, LM.SIG performed well in all cohorts related to SKCM, GBM, RCC, UC, GC and NSCLC, which further demonstrated its vitality in predicting the immunotherapy response in pan-cancer datasets (Fig. 4H, Table S13). Among 7 melanoma-specific signatures, LM.SIG also maintained its position in the top 3, exhibiting an AUC of 0.71 for predicting immunotherapy response in melanoma patients (Figure S2).

### Survival prognostication by LM.SIG

**Fig. 5:**
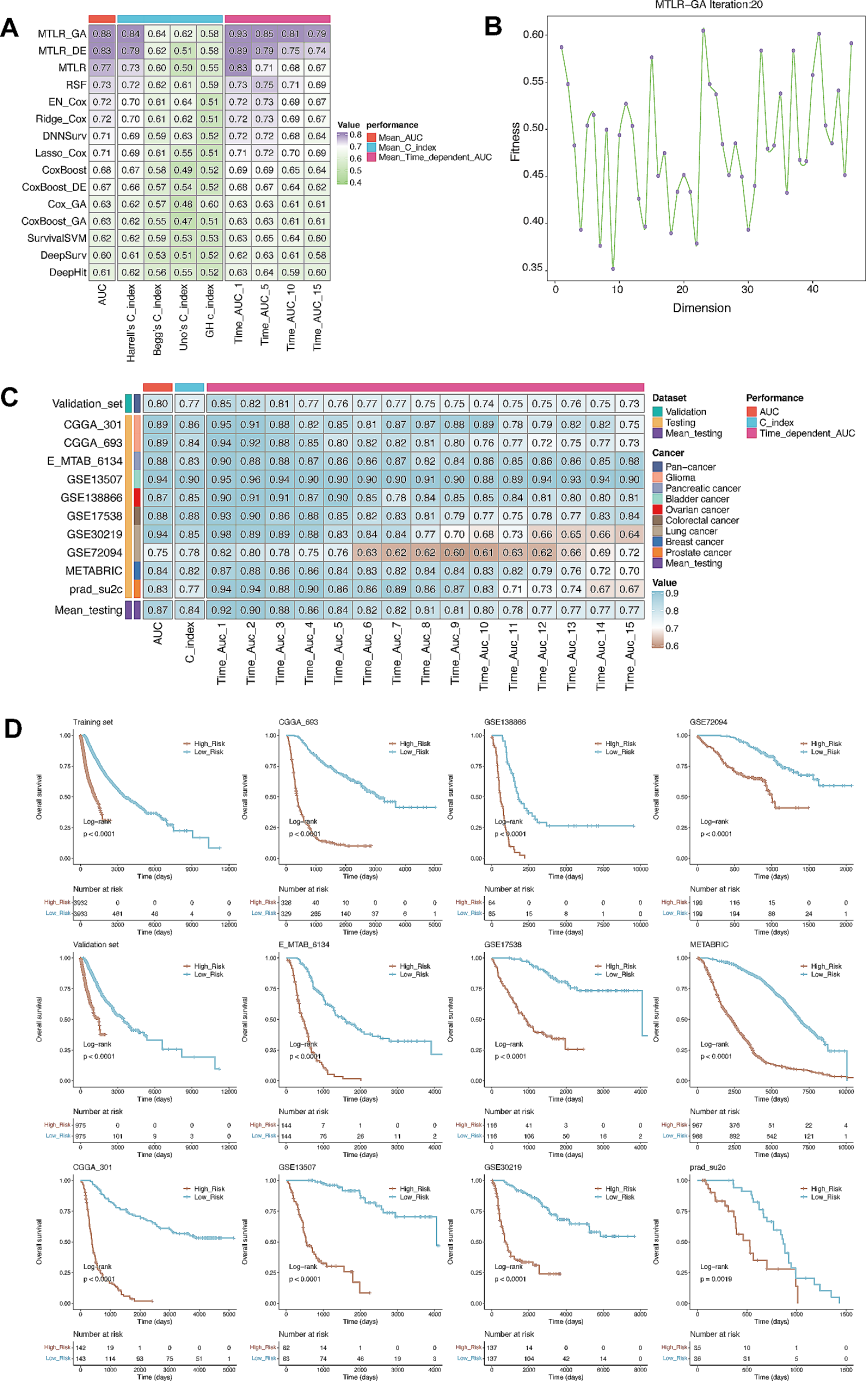
Survival prognostication of LM.SIG. (**A**) Comparison of C-index plots depicting the performance of 15 ML algorithms in the validation set. (**B**) Line Graph illustrating the distribution of model fitness in the MTLR_GA method. (**C**) Comparison of C-index plots depicting the performance of the LM.SIG prognostic model in the validation and testing sets. (**D**) Kaplan‑Meier curves comparing OS between High‑risk and Low‑risk patients in the training, validation and testing sets

To tune the LM.SIG for prognostication of pan-cancer survival, we utilized LM.SIG to develop a survival model based on the SurvBenchmark design [21]. A diverse collection of survival-specific models (including Lasso_Cox, Ridge_Cox, EN_Cox, RSF, MTLR, DNNSurv, CoxBoost, Cox_GA, MTLR_GA, CoxBoost_GA, MTLR_DE, CoxBoost_DE, SurvivalSVM, DeepSurv, DeepHit) were evaluated. We divided the TCGA pan-cancer patients into training set (80%, *n* = 7865) and validation set (20%, *n* = 1950), and trained the LM.SIG prognostic model with training set. Then, the value of AUC, time-dependent AUC and C_index were calculated based on the validation set. As shown in Fig. 5A, MTLR_GA achieved the best performance across the validation set and was identified as the optimal model (Table S14). MTLR_GA represents a multi-task logistic regression model with genetic algorithm as feature selection method. Following the default procedure of processing, genetic algorithm for feature selection was implemented based on the training set via the “GenAlgo” R package. With 20 times iterations, the highest fitness of a solution was deemed as the optimal individual (Fig. 5B). The top 10 genes were finally screened out, including CFH, ECHS1, DNAJC19, IRAK1, LDHA, DGUOK, MRPS28, NDUFB3, NDUFA12 and C1QBP. Based on these 10 hub genes, we trained the training set with the MTLR algorithm using the “MTLR” R package, and the risk score was subsequently calculated for each patient. Meanwhile, the performance of the final model was assessed by the AUC of the validation and testing sets (*n* = 4434). Results illustrated that this LM.SIG related prognostic model achieved excellent performance in validation and testing sets (Fig. 5C). Furthermore, we found that patients with higher risk scores in all enrolled cohorts were associated with worse OS (all *p* < 0.05, Fig. 5D), the same as each dataset in the TCGA pan-cancer cohort (Figure S3). These results led us to conclude that the LM.SIG related prognostic model may stress its potential as a predictive tool in pan-cancer datasets.

### Potential therapeutic targets generated from LM.SIG using CRISPR studies

**Fig. 6:**
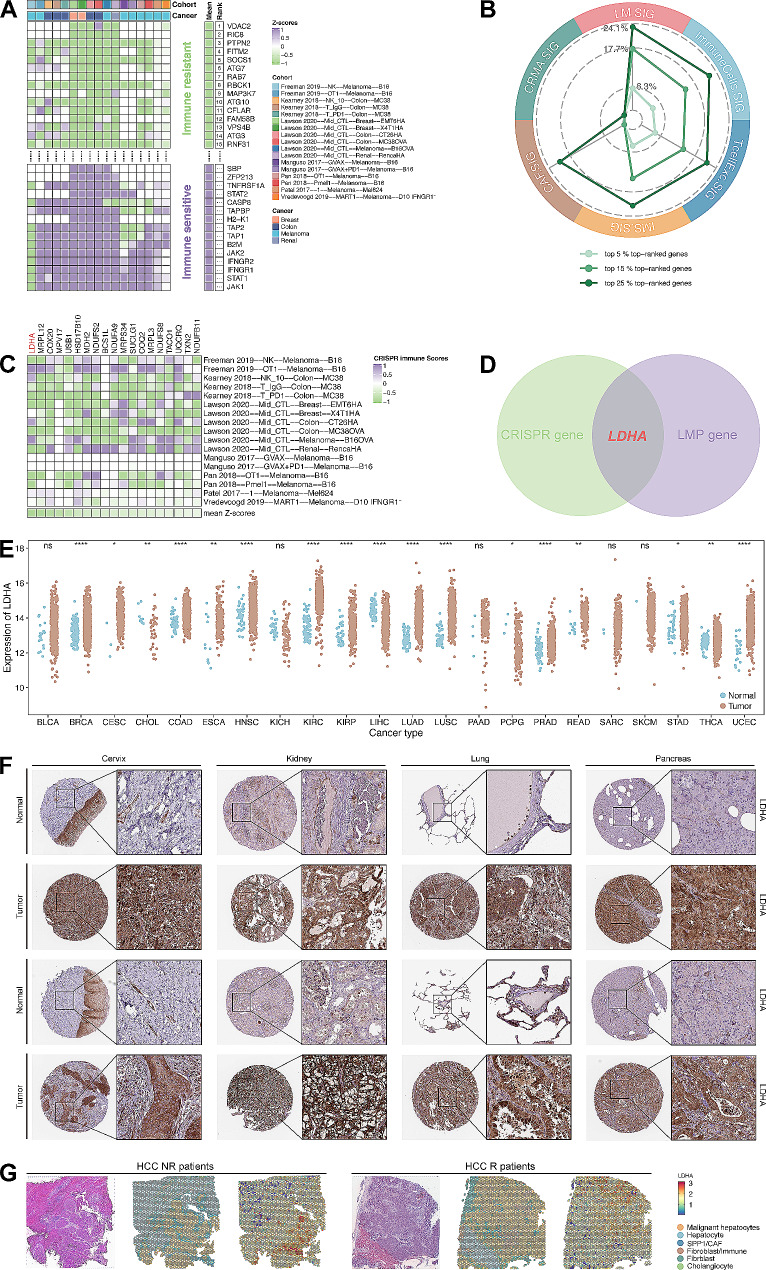
Identification of the potential targets from LM.SIG. (**A**) Ranking of genes based on their knockout effects on anti‑tumor immunity across 17 CRISPR datasets. (**B**) Radar plot comparing the percentage of top‑ranked genes for LM.SIG and other predictive signatures. (**C**) Heatmap depicting z-scores of 19 LM.SIG genes in the 5% top‑ranked genes across different CRISPR datasets. (**D**) Veen plot depicting the intersection of LM.SIG genes ranked in CRISPR datasets and LM prognostic model (LMP gene). (**E**) The expression of LDHA across diverse cancers. (**F**) The representative image of IHC depicted the upregulated protein expression of LDHA in tumor samples of the cervix, kidney, lung and pancreas. **(G)** The expression of LDHA in immunotherapy ST dataset of HCC. (ns, not significant; *p < 0.05, **p < 0.01, ***p < 0.001, ****p < 0.0001)

To facilitate the clinical application of LM.SIG, it’s urgent to identify potential targets in pan-cancer patients. Based on this, 17 CRISPR datasets were split from 7 CRISPR studies with immune response information of knockout genes according to their types of cell lines and treatment conditions. The z-scores of enrolled 22,505 CRISPR genes were ordered and the top-ranked genes were regarded as immune-resistant genes (Fig. 6A, Table S15). In other words, genes ranked top may elevate the capacity of anti-tumor immunity after knockout, whereas those bottom-ranked genes may restrain anti-tumor immunity after knockout. Then, we compared the percentage of top-ranked genes in LM.SIG and those in published immune-resistant signatures (including TcellExc.SIG [40], ImmuneCells.Sig [36], IMS.SIG [42], CAF.SIG [36], and CRMA.SIG [39]). As shown in Fig. 6B, LM.SIG accounted for the highest proportion of top-ranked genes than other signatures. Interestingly, the top 5% of genes (*n* = 19) were over-represented in LM.SIG (Fisher’s exact test, *p* = 0.04). These genes were further validated in multiple independent CRISPR datasets and served as potential targets with immunotherapy (Fig. 6C, Table S16).

To further investigate the targets with both immunotherapy prediction and survival prognostication, we intersected the 19 genes selected in CRISPR datasets and 10 genes enrolled in LM.SIG related prognostic model (LMP genes). Ultimately, LDHA, the top-ranked gene of LM.SIG in multiple CRISPR datasets, was identified as the hub gene (Fig. 6D). As shown in Fig. 6E, among 17/22 (77.27%) TCGA pan-cancer datasets, the expression of LDHA was upregulated in tumor samples compared to normal samples. The IHC in Human Protein Atlas (HPA) datasets also revealed the elevated protein expression level of LDHA in cervical cancer, kidney cancer, lung cancer and pancreatic cancer (Fig. 6F). Besides, two immunotherapy scRNA-seq cohorts (GSE115978, GSE123813) and one ST immunotherapy cohort revealed that upregulated LDHA mainly enriched in NR compared to R or TN patients at both single-cell (Figure S4) and spatial levels (Fig. 6G). In conclusion, LDHA from derived from LM.SIG might serve as a prognostic pan-cancer biomarker that also predicts immunotherapy response.

### LDHA deficiency enhanced the anti-tumor immunity and immunotherapy response in pancreatic cancer

**Fig. 7:**
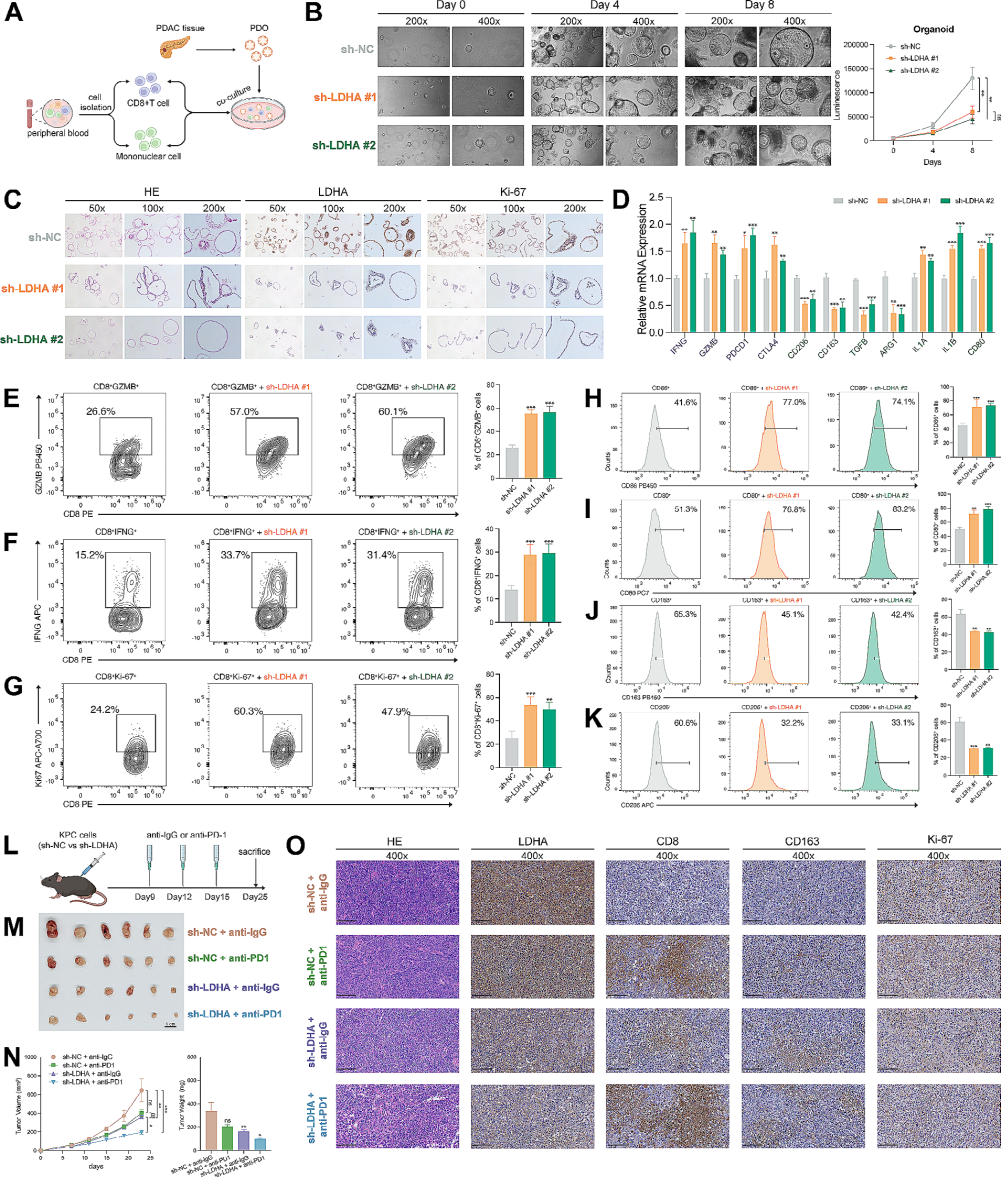
Validation of the role of LDHA in pancreatic cancer. (**A**) Workflow displaying the immune cells co-cultured with PDOs. (**B**) Microscopic observation (left panel) and CTG assay (right panel) demonstrated the knockdown of LDHA significantly slowed the growth rate of PDOs. (**C**) IHC staining showing the expression of LDHA and Ki-67 in sh-NC and sh-LDHA groups. (**D**) RT-qPCR revealed the expression of candidate markers in sh-NC and sh-LDHA PDOs. Representative plots and percentages of CD8^+^GZMB^+^ (**E**), CD8^+^IFNG^+^ (**F**) and CD8^+^Ki-67^+^ (**G**) cells from sh-NC and sh-LDHA groups. Representative histograms and percentages of CD86^+^ (**H**), CD80^+^ (**I**), CD163^+^ (**J**) and CD206^+^ (**K**) cells from sh-NC and sh-LDHA groups. (**L**) Workflow of the in vivo experiments. (**M**) Representative images of subcutaneous PC tumors from mice treated with sh-NC + anti-IgG, sh-NC + anti-PD1, sh-LDHA + anti-IgG and sh-LDHA + anti-PD1. (**N**) The growth rate (left panel) and weight (right panel) of tumors in response to the treatment of sh-NC + anti-IgG, sh-NC + anti-PD1, sh-LDHA + anti-IgG and sh-LDHA + anti-PD1. (**O**) IHC staining showing the expression of LDHA, CD8, CD163 and Ki-67 among sh-NC + anti-IgG, sh-NC + anti-PD1, sh-LDHA + anti-IgG and sh-LDHA + anti-PD1 groups (scale bar: 100 μm). (ns, not significant; *p < 0.05, **p < 0.01, ***p < 0.001, ****p < 0.0001)

To further validate the role of LDHA in cancer immunotherapy, we conducted the following experiments in the realm of PC. Firstly, we found that the knockdown of LDHA significantly suppressed lactate levels of PC PDOs (Figure S5A-C). Then, CD8^+^ T cells and CD14^+^ mononuclear macrophages were isolated from human peripheral blood, activated in vitro and co-cultured with PC PDOs (Fig. 7A). Microscopic observation and CTG assay showed that knockdown of LDHA significantly slowed the growth rate of PDOs (Fig. 7B). Lower expression of Ki-67 was also observed in sh-LDHA PDOs by IHC staining (Fig. 7C). Meanwhile, CD8^+^ T cells and macrophages in the co-culture system were analyzed. The results of RT-qPCR showed that the expression of killer genes and immune checkpoint genes of T cells (IFNG, GZMB, PDCD1 and CTLA4) was significantly elevated when co-cultured with PDOs with sh-LDHA, while the expression of M2-related markers of macrophages (CD206, CD163, TGFB and ARG1) was decreased and the expression of M1-related markers (IL1A, IL1B and CD80) was increased (Fig. 7D). These results indicated a switch from protumoral to antitumoral macrophages in sh-LDHA PDOs. Furthermore, flow cytometry analysis showed that CD8^+^ T cells in the sh-LDHA groups had a stronger capacity for anti-tumor activity and proliferation (Fig. 7E-G), and macrophages were more inclined to antitumoral M1 polarization (Fig. 7H-K) than those in the sh-NC group. Next, we generated the KPC subcutaneous tumors in C57 mice and injected anti-IgG or anti-PD1 on days 9,12 and 15 intraperitoneally (Fig. 7L). Compared with the sh-NC group, the effect of anti-PD1 treatment in the sh-LDHA group was more significant, and the tumor reduction was more obvious (Fig. 7M, N). IHC results also demonstrated that tumors with sh-LDHA had abundant immune cell infiltration and slower tumor proliferation (Fig. 7O, Figure S6). In conclusion, our experimental results suggested that LDHA affected the state of immune cells in TIME and low expression of LDHA increased their efficacy in immunotherapy of PC.

## Discussion

Accumulating data have revealed that LM is a vital part of aberrant cancer metabolism and contributes to immunosuppression in the TME [17, 63, 64], but direct evidence concerning the connection between LM and the response to immunotherapy is absent. In this study, we employed the GSVA method to assess the LM of malignant cells and demonstrated the negative relationship between LM and immunotherapy response based on two immunotherapy scRNA-seq cohorts of SKCM and BCC. These results inspired us to further hypothesize that there exists a negative relationship between LM and immunotherapy response across pan-cancer cohorts. Then, an extensive in-depth analysis was conducted to screen out upregulated genes in malignant cells that related to elevated LM in 40 scRNA-seq cohorts. These genes represent a pan-caner LM-related signature and were named LM.SIG. In addition, rigorous validation of LM.SIG revealed its superior predictive performance for immunotherapy response compared to previous signatures across multiple independent immunotherapy bulk RNA-Seq cohorts. Our study stands as the inaugural report demonstrating the robust link between LM and immunotherapy outcomes via a meticulous analysis of extensive datasets.

We noticed that LM.SIG genes were mainly enriched in NADH-related and TCA cycle-related pathways. Quinn et al. elucidated that lactate exerts an acidity-independent suppressive influence on the T cells immunotherapy via a transition from NAD^+^ to NADH which is known as lactate-induced reductive stress [16]. TCA cycle, a critical metabolic pathway underlying tumor cell metabolism for energy production to the cell, is reported to be a novel immune checkpoint blockade as inhibiting it could improve the anti-PD-1 immunotherapy efficacy in melanoma cells via ATF3-mediated PD-L1 expression and glycolysis [65]. Our findings aligned with prior research and indicated that LM.SIG incorporates genes exhibiting robust and specific correlations with immunotherapy.

The following transcriptomic analysis in 30 types of cancer obtained from TCGA datasets illustrated the decreased expression of immune-related genes and abundance of immune cells in samples with high LM.SIG. With regards to these immune cells, elevated lactate levels hinder the antitumor efficacy of T cells by promoting H^+^ accumulation and sustaining a low pH within the TME and acidic TME can enhance the suppression of antitumor immunity, thereby reducing the effectiveness of immunotherapy [66]. Under the circumstance of lactic acidosis, monocytes could differentiate into either dendritic cells with an immunosuppressive phenotype [67] or macrophages with an inflammatory protumor phenotype [68]. Subsequent analysis revealed immune-suppressive pathways positively correlated with LM.SIG across pan-cancer datasets, including metabolism, DNA repair, and MYC signaling. Jaiswal AR et al. revealed that melanoma can evade T-cell checkpoint blockade immunotherapy by adapting a hypermetabolic phenotype [69]. DNA repair was confirmed to be a predictor for anti-PD-1/PD-L1 immunotherapy efficacy in tumor [70]. Besides, the activated MYC pathway inhibited the immunotherapy response by upregulating the expression of PD-L1 and CD47 [71]. Also, we divided pan-cancer patients into 4 subgroups (HLHT, HLLT, LLHT and LLLT) according to the level of TMB and LM.SIG. TMB is widely acknowledged as an immunotherapy response biomarker, higher TMB indicates better immunotherapy response. Our stratified analysis also revealed the LLHT subgroup possessed the strongest capacity of anti-tumor immunity. All these results demonstrated the immunosuppressive characteristic of high LM.SIG levels, implying the predictive value of LM.SIG.

With the assistance of the optimal NB algorithm, LM.SIG was identified as a novel signature that is proficient in predicting immunotherapy response across various cancer types, including RCC, UC, SKCM and GBM. To substantiate its efficacy, LM.SIG was systematically compared with other leading-edge signatures (6 pan-cancer signatures). LM.SIG demonstrated superior performance, surpassing pan-cancer signatures with enhanced generalization and exhibiting consistently favorable outcomes across diverse cohorts spanning multiple cancer types. To further enhance the prognostic efficacy of the LM.SIG, MTLR_GA in SurvBenchmark design was applied on TCGA pan-cancer datasets. Our findings revealed that patients with higher risk scores tended to obtain miserable OS. Together, we demonstrated that the LM.SIG performed excellently in immunotherapy prediction and survival prognostication across pan-cancer patients.

Owing to the distinguished performance of LM.SIG in predicting immunotherapy outcomes, there is an urgent need to identify potential targets from LM.SIG. Hence, CRISPR datasets were utilized and we ranked the genes based on their logFCs of sgRNA reads in immune-competent or -deficient conditions. After intersecting top-ranked genes in CRISPR datasets and top genes selected in LM.SIG related prognostic model, we identified LDHA as the hub gene for further analysis. LDHA catalyzes the conversion of pyruvate to lactate, concurrently oxidizing NADH to NAD^+^ [72]. Previous studies revealed that LDHA plays a vital role in tumor biology, including initiation, development, progression, invasion, metastasis, angiogenesis, and immune evasion [73], and targeting LDHA is considered a safe therapeutic strategy. In addition, we validated the mRNA and protein expression of LDHA in TCGA datasets and HPA datasets, respectively. The immunotherapy cohorts of scRNA-seq and ST data were also collected to indicate the negative correlation between the expression of LDHA and immunotherapy response. Furthermore, we illustrated that the downregulation of LDHA in pancreatic cancer heightened the antitumor immune response of CD8^+^ T cells and enhanced the antitumoral polarization of macrophages. Consequently, this augmentation resulted in an improved effectiveness of immunotherapy. In summary, LDHA emerges as a plausible therapeutic target across diverse cancer types, and the other top-ranked genes are imperative for advancing the development of a comprehensive immunotherapeutic strategy.

Several limitations should be mentioned in our study. First, there were only TN and NR patients in GSE115978. Given the recognized average response rate of melanoma at 30–40%, a notable proportion of TN patients might not respond to immunotherapy. Theoretically, the disparity between TN and NR subgroups is expected to be less pronounced than that between R and NR subgroups, as TN comprises a mixture of NR and R. However, our study revealed a substantial difference in LM levels between NR and TN, suggesting a more significant gap between NR and R. This observation was further validated through the analysis of another scRNA-seq cohort, GSE123813. Additionally, the predictive performance of LM.SIG and LDHA at the pan-cancer level should be further investigated via more experiments.

## Conclusions

In conclusion, our investigations yield innovative insights into diverse molecular and metabolic processes linked to LM in the immunotherapy response prediction and survival prognostication. Through a comprehensive pan-cancer analysis of single-cell and bulk transcriptomic data, we formulated an LM.SIG and validated its competence to predict immunotherapy and prognostic outcomes across diverse cohorts. Subsequent investigation into LM.SIG identified the LDHA as the most potential therapeutic target in pancreatic cancer. Our study suggested a new direction for improving the effectiveness of tumor immunotherapy by targeting metabolic reprogramming.

### Electronic supplementary material

Below is the link to the electronic supplementary material.

Supplementary Material 1: Figure S1. AUC value of LM.SIG in three independent testing cohorts. Figure S2. Bar plot depicting the AUC values of LM.SIG and other melanoma‑specific signatures in the SKCM cohort (Hugo 2016 + Van Allen 2015). Figure S3. The association between LM.SIG-related risk score and OS of patients in each TCGA pan-cancer dataset (all *p* < 0.05). Figure S4. The expression of LDHA in GSE115978 and GSE123813 datasets. Figure S5. The level of lactate in sh-LDHA PDOs. The mRNA (A) and protein (B) levels of LDHA in sh-LDHA PDOs. (C) The level of lactate in sh-LDHA PDOs. (ns, not significant; *p < 0.05, **p < 0.01, ***p < 0.001, ****p < 0.0001) Figure S6. IHC staining showing the expression of LDHA, CD8, CD163 and Ki-67 among sh-NC + anti-IgG, sh-NC + anti-PD1, sh-LDHA + anti-IgG and sh-LDHA + anti-PD1 groups (scale bar: 200 μm).

Supplementary Material 2: Table S1. Characteristics of selected patients in 2 immunotherapy scRNA-seq cohorts and 1 ST cohort. Table S2. List of scRNA datasets applied to develop LM.SIG. Table S3. List of pan-cancer transcriptomic datasets. Table S4. List of bulk RNA-seq immunotherapy datasets. Table S5. List of CRISPR datasets. Table S6. List of LM-related genes. Table S7. ML algorithms in SurvBenchmark design. Table S8. The forward and reverse primers in RT-qPCR analysis. Table S9. List of *LM*_*x*_ genes in each scRNA-seq dataset. Table S10. List of *LM*_*y*_ genes in each scRNA-seq dataset. Table S11. List of *LM*_*n*_ genes in each scRNA-seq dataset. Table S12. List of LM.SIG genes. Table S13. Comparison of AUC between LM.SIG and other well-established signatures. Table S14. Summary for computational efficiency of 15 ML algorithms. Table S15. List of enrolled 22,505 CRISPR genes in 17 datasets. Table S16. List of the top 5% of CRISPR genes over-represented in LM.SIG.

## Data Availability

Data are available in a public, open-access repository. Essential scripts for the construction of LM.SIG are available on the GitHub website (https://github.com/Dongjie-orange/Pancancer_LM_SIG).

## Abbreviations

AdaBoost: AdaBoost Classification Trees
AUC: Area Under Curve
BCC: Basel Cell Carcinoma
B-H: Benjamini-Hochberg procedure
BRCA: Breast Cancer
CHOL: Cholangiocarcinoma
C-index: concordance index
CoxBoost_DE: Boosting cox model with ranking based method as feature selection method
CoxBoost_GA: Boosting cox model with genetic algorithm as feature selection method
Cox_GA: Cox model with genetic algorithm as feature selection method
CRC: Colorectal Cancer
DLBC: diffuse large B cell lymphoma
DNNSurv: Deep learning survival model
EGA: European Genome-phenome Archive
EN_Cox: Elastic net cox
FDR: false discovery rate
GBM: Glioblastoma Multiforme
GC: Gastric Cancer
GEO: Gene Expression Omnibus
GO: Gene Ontology
GSVA: Gene set variation analysis
HCC: hepatocellular carcinoma
H&E: hematoxylin and eosin
HNSCC: Head and Neck Squamous Cell Carcinoma
HPA: Human Protein Atlas
HSCLC: Non-small Cell Lung Cancer
IHC: immunohistochemistry
KEGG: Kyoto Encyclopedia of Genes and Genomes
KNN: k-nearest neighbors
LAML: acute myeloid leukemia
LIHC: Liver Hepatocellular Carcinoma
LM: lactate metabolism
LM.SIG: LM-related signature
logFCs: Log-fold changes
LogiBoost: boosted logistic regressions
MB: Medulloblastoma
MCC: Merkel Cell Carcinoma
ML: machine learning
MM: Multiple Myeloma
MsigDB: Molecular Signatures Database
MTLR: Multi-task logistic regression
MTLR_DE: Multi-task logistic regression model with ranking based method as feature selection method
MTLR_GA: Multi-task logistic regression model with genetic algorithm as feature selection method
NADH: Nicotinamide adenine dinucleotide
NB: Naïve Bayes
NET: Neuroendocrine Tumor
NR: non-responders
OS: overall survival
OV: Ovarian Cancer
PAAD: Pancreatic Adenocarcinoma
PBMCs: Peripheral Blood Mononuclear Cells
PC: Pancreatic Cancer
PDOs: Patient-derived organoids
R: responders
RF: random forest
RSF: Random survival forest
scRNA-seq: single-cell RNA-Seq
sgRNA: small guide RNA
SKCM: Skin Cutaneous Melanoma
ST: spatial transcriptomics
STAD: Stomach Adenocarcinoma
SVM: support vector machine
SurvivalSVM: Survival support vector machine
TCA: tricarboxylic acid
TCGA: The Cancer Genome Atlas
THYM: thymoma
TILs: tumor-infiltrating leukocytes
TMB: tumor mutation burden
TME: tumor microenvironment
TN: treatment-naïve
t-SNE: t-distributed Stochastic Neighbor Embedding
UVM: Uveal melanoma
