# Supplementary material for: Pan-cancer analysis implicates novel insights of lactate metabolism into immunotherapy response prediction and survival prognostication
Source: J Exp Clin Cancer Res. 2024 Apr 25;43:125. doi: 10.1186/s13046-024-03042-7 (PMC11044366; doi:10.1186/s13046-024-03042-7)
Supplement: Supplementary file 1 — Supplementary Material 1: Figure S1. AUC value of LM.SIG in three independent testing cohorts. Figure S2. Bar plot depicting the AUC values of LM.SIG and other melanoma‑specific signatures in the SKCM cohort (Hugo 2016 + Van Allen 2015). Figure S3. The association between LM.SIG-related risk score and OS of patients in each TCGA pan-cancer dataset (all p < 0.05). Figure S4. The expression of LDHA in GSE115978 and GSE123813 datasets. Figure S5. The level of lactate in sh-LDHA PDOs. The mRNA (A) and protein (B) levels of LDHA in sh-LDHA PDOs. (C) The level of lactate in sh-LDHA PDOs. (ns, not significant; *p < 0.05, **p < 0.01, ***p < 0.001, ****p < 0.0001) Figure S6. IHC staining showing the expression of LDHA, CD8, CD163 and Ki-67 among sh-NC + anti-IgG, sh-NC + anti-PD1, sh-LDHA + anti-IgG and sh-LDHA + anti-PD1 groups (scale bar: 200 μm). [file 13046_2024_3042_MOESM1_ESM.pdf]

# Supplementary Figure 1

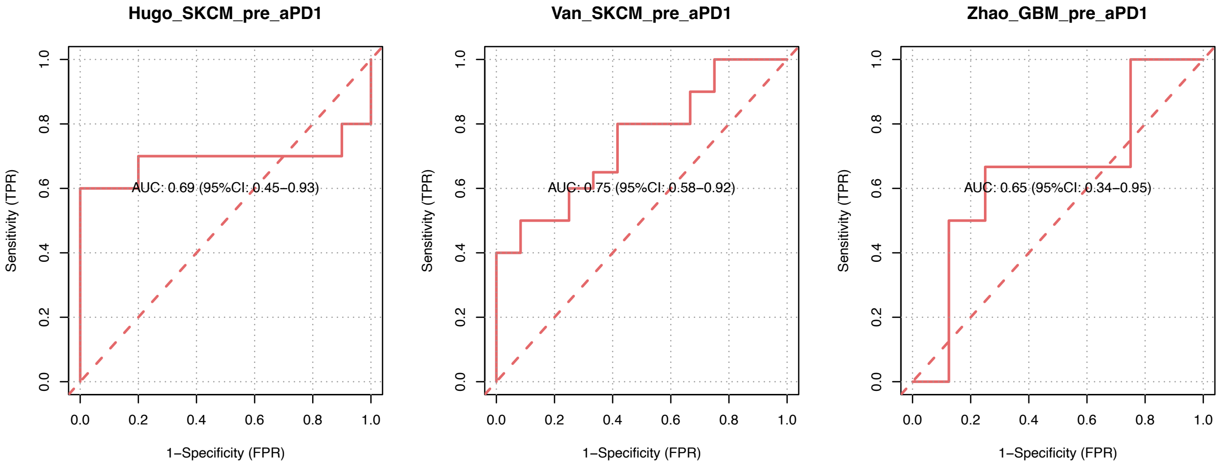


**Figure S1. AUC value of LM.SIG in three independent testing cohorts.**

# Supplementary Figure 2

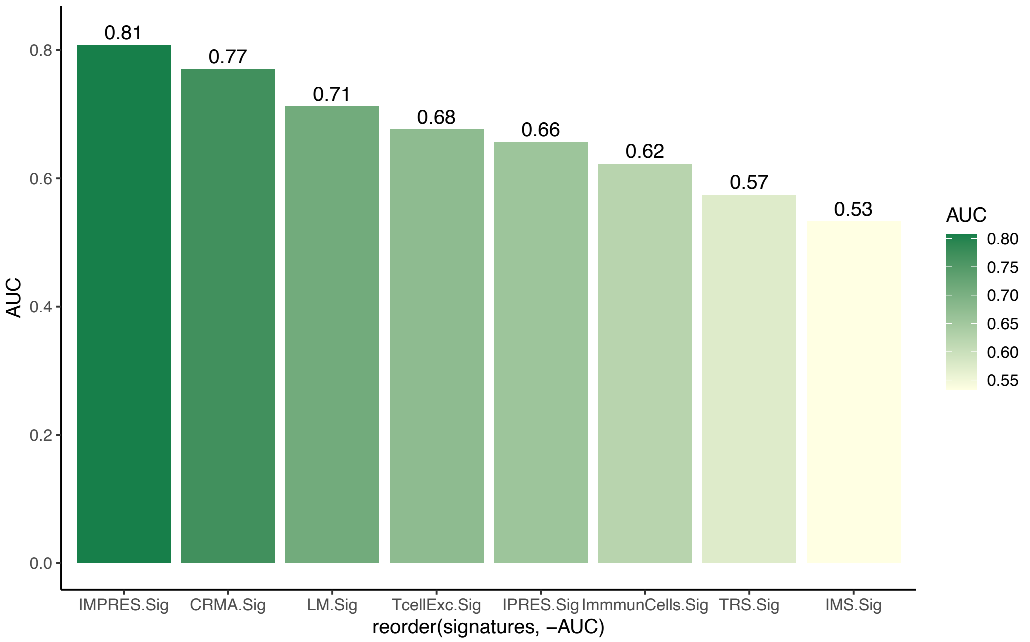


**Figure S2. Bar plot depicting the AUC values of LM.SIG and other melanoma-specific signatures in the SKCM cohort (Hugo 2016 + Van Allen 2015).**

# Supplementary Figure 3

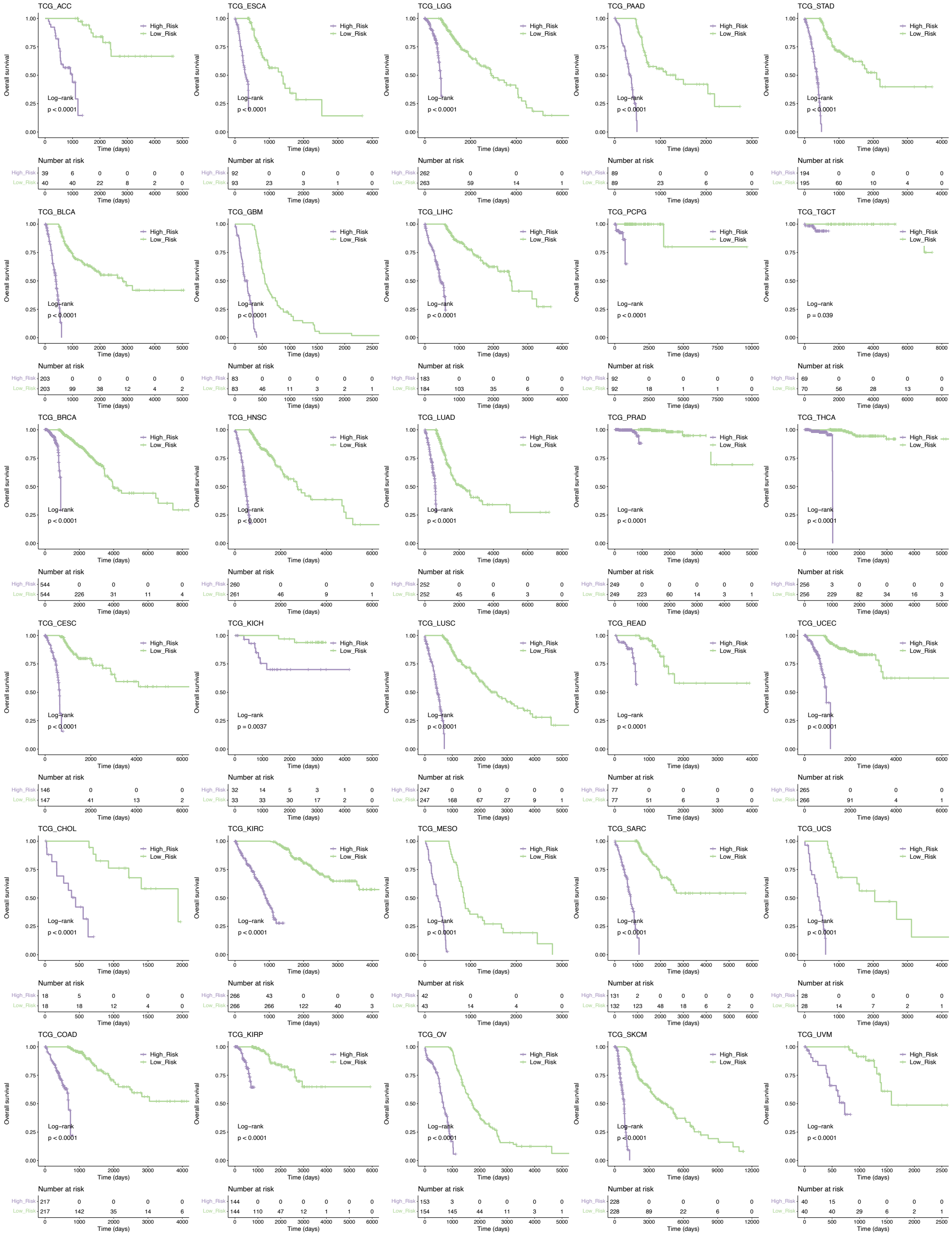


**Figure S3. The association between LM.SIG-related risk score and OS of patients in each TCGA pan-cancer dataset (all p < 0.05).**

# Supplementary Figure 4

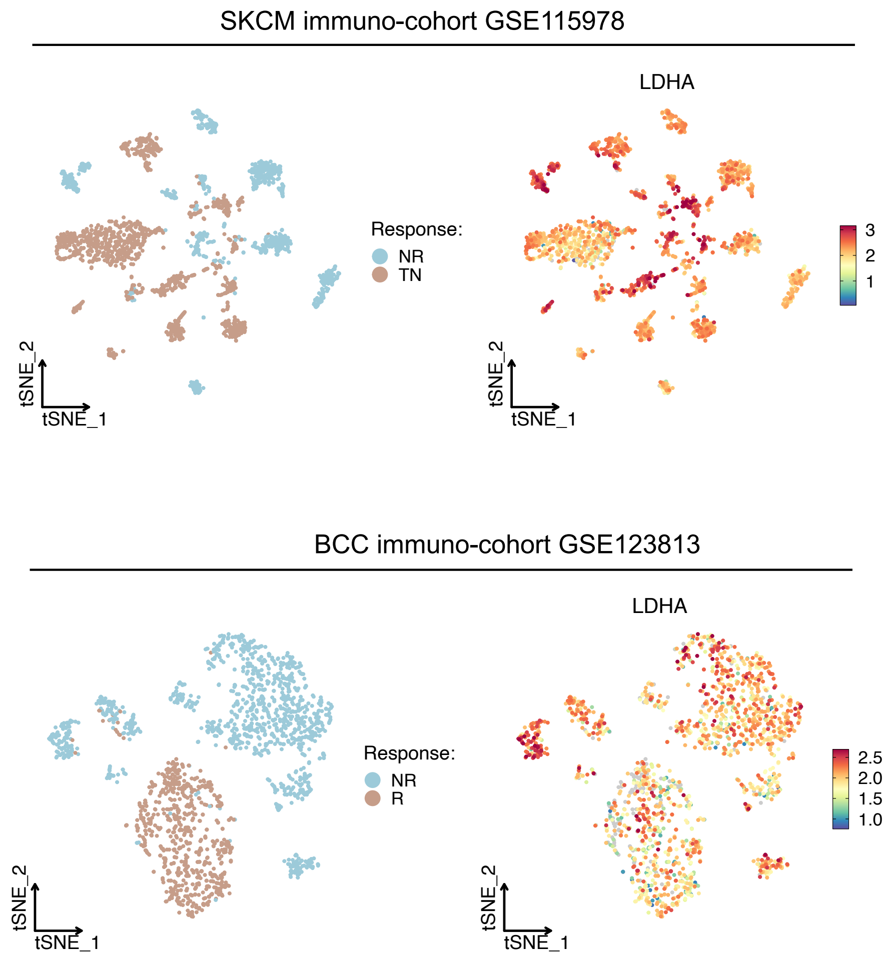


**Figure S4. The expression of LDHA in GSE115978 and GSE123813 datasets.**

# Supplementary Figure 5

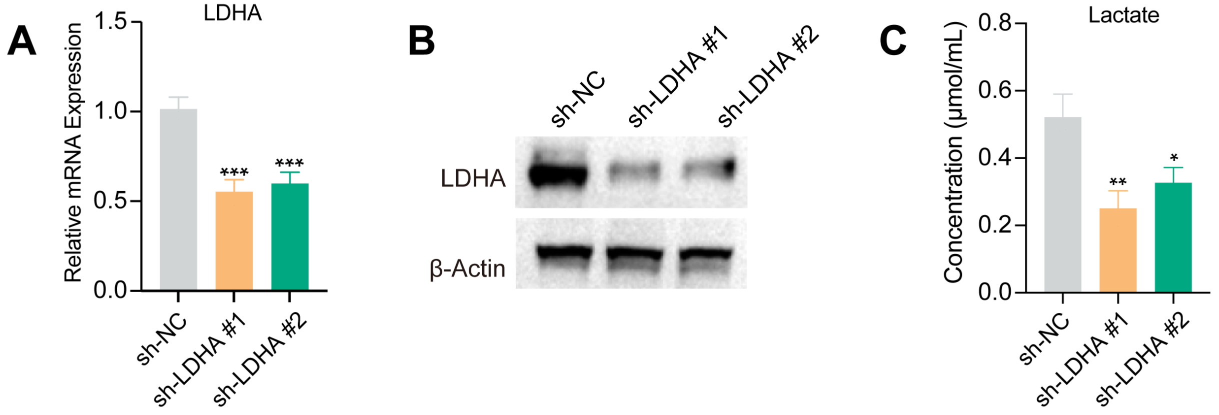


**Figure S5. The level of lactate in sh-LDHA PDOs.** The mRNA (**A**) and protein (**B**) levels of LDHA in sh-LDHA PDOs. (**C**) The level of lactate in sh-LDHA PDOs. (ns, not significant; *$p < 0.05$, **$p < 0.01$, ***$p < 0.001$, ****$p < 0.0001$)

# Supplementary Figure 6

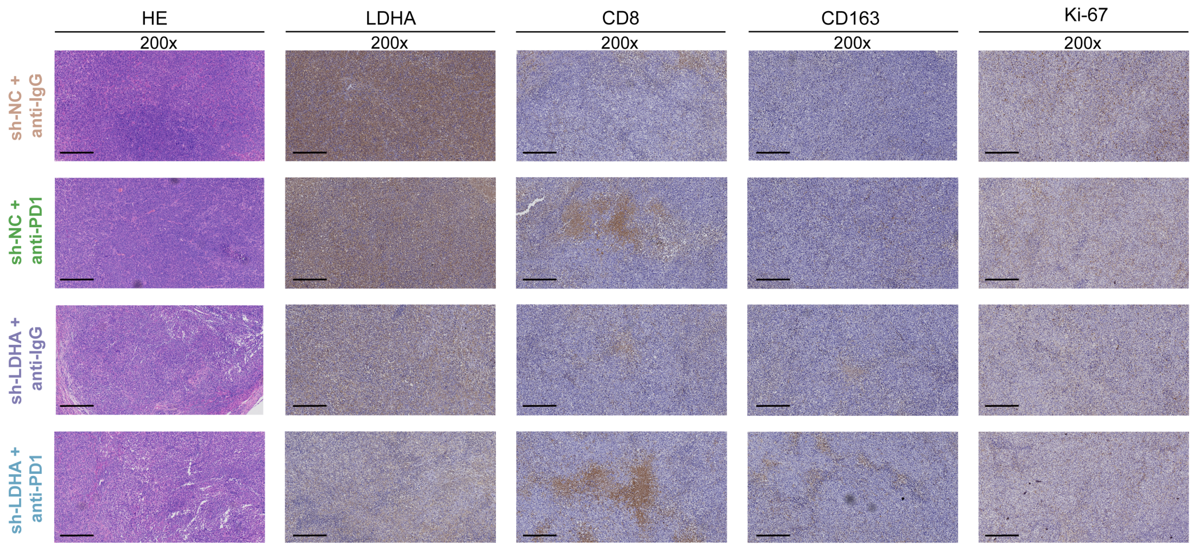


**Figure S6. IHC staining showing the expression of LDHA, CD8, CD163 and Ki-67 among sh-NC+anti-IgG, sh-NC+anti-PD1, sh-LDHA+anti-IgG and sh-LDHA+anti-PD1 groups (scale bar: 200 μm).**
